# Miltiradiene Production by Cytoplasmic Metabolic Engineering in *Nicotiana benthamiana*

**DOI:** 10.3390/metabo13121188

**Published:** 2023-12-06

**Authors:** Xiangxiang Ren, Chuhang Lin, Yanbo Huang, Tao Su, Juan Guo, Lei Yang

**Affiliations:** 1 Co-Innovation Center for Sustainable Forestry in Southern China, College of Life Sciences, Nanjing Forestry University, Nanjing 210037, China; renxiangxiang@njfu.edu.cn (X.R.); sutao@njfu.edu.cn (T.S.); 2 Shanghai Key Laboratory of Plant Functional Genomics and Resources, Shanghai Chenshan Botanical Garden, Shanghai 201602, China; linchuhang@csnbgsh.cn (C.L.); huangyanbo@csnbgsh.cn (Y.H.); 3 State Key Laboratory of Dao-Di Herbs, Beijing 100700, China; guojuan@wbgcas.cn

**Keywords:** miltiradiene, plant synthetic biology, *Nicotiana benthamiana*, tanshinone biosynthesis pathway

## Abstract

Plant natural products are important sources of innovative drugs, but the extraction and isolation of medicinal natural products from plants is challenging as these compounds have complex structures that are difficult to synthesize chemically. Therefore, utilizing heterologous expression systems to produce medicinal natural products in plants is a novel, environmentally friendly, and sustainable method. In this study, *Nicotiana benthamiana* was used as the plant platform to successfully produce miltiradiene, the key intermediate of tanshinones, which are the bioactive constituents of the Chinese medicinal plant *Salvia miltiorrhiza*. The yield of miltiradiene was increased through cytoplasmic engineering strategies combined with the enhancement of isoprenoid precursors. Additionally, we discovered that overexpressing *SmHMGR* alone accelerated apoptosis in tobacco leaves. Due to the richer membrane systems and cofactors in tobacco compared to yeast, tobacco is more conducive to the expression of plant enzymes. Therefore, this study lays the foundation for dissecting the tanshinone biosynthetic pathway in tobacco, which is essential for subsequent research. Additionally, it highlights the potential of *N. benthamiana* as an alternative platform for the production of natural products in plants.

## 1. Introduction

Danshen (*Salvia miltiorrhiza* Bunge) is a perennial medicinal herb belonging to the family Lamiaceae [1]. The dried root and rhizome of *S. miltiorrhiza* have a well-established history in traditional Chinese medicine and are frequently utilized. This usage can be traced back to the ancient Chinese pharmacy monograph “Shennong Materia Medica” [2]. *S. miltiorrhiza* is known for its effects in promoting blood circulation, relieving pain, regulating menstruation, and calming the mind [3]. Modern pharmacological research indicates that *S. miltiorrhiza* contains a rich array of constituents, including salvianolic acid, tanshinones, volatile oils, and sugars [4]. Among these, tanshinones are the major bioactive compounds responsible for *S. miltiorrhiza*’s therapeutic effects, which find extensive application in the treatment of tumors [5], diabetes [6], and cardiovascular and cerebrovascular diseases [7]. Recent studies have further revealed the potential use of tanshinone IIA in cancer therapy [8]. Given the medicinal value of tanshinones, there is a growing demand for *S. miltiorrhiza*. However, the availability of arable land and suitable cultivation conditions are limited, necessitating continuous cropping to ensure *S. miltiorrhiza*’s yield [9]. Nevertheless, this practice is prone to encountering continuous cropping obstacles. Continuous cropping triggers severe soil-borne diseases, leading to decreased crop yield and quality, thereby impeding the sustainable development of agriculture [10]. Particularly in the case of medicinal plants, it has been reported that approximately 70% of those used for their roots and rhizomes experience varying degrees of continuous cropping obstacles [11]. Additionally, due to the unclear causes of continuous cropping obstacles, many farmers blindly intensify fertilizer and pesticide usage to maintain crop yield. This not only fails to effectively address the issue but also increases production costs and results in soil degradation and excessive pesticide residue issues [12]. Synthetic biology provides new ideas and approaches for the production of natural medicinal compounds in plants [13]. The application of synthetic biology in the field of plant natural products mainly includes two aspects: one is to directly promote the synthesis of natural medicinal compounds in plants through genetic engineering and metabolic engineering [14], and the other is to reconstruct or optimize the biosynthetic pathways of natural medicinal compounds and synthesize target compounds in microbial or plant chassis cells [15]. Therefore, unraveling the biosynthetic pathway of tanshinones and using synthetic biology methods to select efficient host heterologous production holds important scientific significance and practical value.

**Figure 1:**
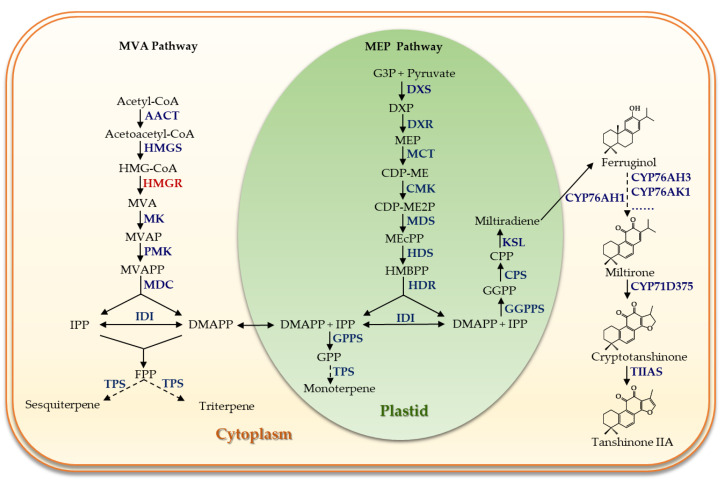
Terpenoid biosynthesis pathway in *Salvia miltiorrhiza* Bung. AACT, acetoacetyl-CoA thiolase; HMGS, 3-hydroxy-3-methylglutaryl-CoA synthase; HMGR, HMG-CoA reductase; MK, mevalonate kinase; PMK, phosphomevalonate kinase; MDC, mevalonate diphosphate decarboxylase; DXS, 1-deoxy-D-xylulose 5-phosphate synthase; G3P, glycer-aldehyde-3-phosphate; DXP, 1-deoxy-D-xylulose 5-phosphate; MEP, 2-C-methyl-D-erythritol 4-phosphate; DXR, 1-deoxy-D-xylulose-5-phosphate reductoisomerase; DXP, 1-deoxy-D-xylulose-5-phosphate; CDP-ME, 4-diphosphocytidyl-2-C-methyl-D-erythritol; MCT, MEP cytidyl-transferase; CMK, 4-(cytidine5-diphospho)-2-C-methylerythritol kinase; CDP-ME2P, 4-diphosphocytidyl-2-C-methyl-D-erythritol 2-phosphate; MEcPP, 2-C-methyl-D-erythritol 2,4-cyclodiphosphate; MDS, 2-C-methyl-D-erythritol 2,4-cyclodiphosphate synthase; HMBPP, (E)-4-hydroxy-3-methyl-but-2-enyl diphosphate; HDS, hydroxy-methybutenyl-4-diphosphate synthase; HDR, 1-hydroxy-2-methyl-2-(E)-butenyl-4-diphosphate reductase; IPP, isopentenyl diphosphate; GPP, geranyl diphosphate; GPPS, geranyl diphosphate synthase; FPP, farnesyl diphosphate; FPPS, farnesyl diphosphate synthase; GGPP, geranylgeranyl diphosphate; GGPPS, geranylgeranyl diphosphate synthase.

Tanshinones, the most abundant lipid-soluble bioactive constituents of *S. miltiorrhiza*, are a class of structural, highly oxidized, abietane-type diterpenoids [16]. The biosynthesis of tanshinones can be broadly divided into three stages (Figure 1). Firstly, the precursors for all terpenoids, isopentenyl diphosphate (IPP) and dimethylallyl pyrophosphate (DMAPP), are produced via the mevalonate (MVA) pathway in the cytoplasm and the methylerythritol phosphate (MEP) pathway in plastids [17]. Subsequently, IPP and DMAPP in plastids are converted to geranylgeranyl diphosphate (GGPP) under the action of geranylgeranyl diphosphate synthase (GGPPS) [18]. This GGPP is then protonated and cyclized to form copalyl diphosphate ((+)-CPP) by the class II diterpene synthase, copalyl diphosphate synthase (CPS) [19]. The ionization-initiated cyclization of (+)-CPP is catalyzed by the class I diterpene synthase, kaurene synthase-like (KSL), to generate the carbon skeleton structure of a diterpenoid, namely miltiradiene [20]. Finally, miltiradiene is transported to the cytoplasm, where it undergoes a series of oxidation modifications by various cytochrome P450s (CYPs) to yield various tanshinones [21].

Miltiradiene is the initial step in the biosynthesis of tanshinones and serves as a crucial intermediate for the biosynthesis of many pharmacologically active natural diterpene compounds besides tanshinone, such as triptolide, carnosic acid, carnosol, and rubesanolides A-D [22,23,24,25,26,27,28,29,30]. Hu, T et al. successfully engineered the yeast strain by introducing the *SmCPS* and *SmKSL* genes and cultivated it in a 5 L fermentation tank, achieving a miltiradiene production of 3.5 g/L [31]. Although successful production of miltiradiene has been achieved in yeast engineered strains, there remain differences between yeast cells and plant cells [32]. When exploring the functions of unknown downstream genes, plant cells have more extensive membrane systems and cofactors, which provide a more suitable enzymatic reaction environment [33]. *N. benthamiana*, as a model plant, possesses advantages such as a fast growth rate, strong environmental adaptability, and low cultivation cost [34]. Among them, *Agrobacterium*-mediated transient transformation in tobacco is widely used in the research of plant gene function due to its ease of operation, short cycle, and high expression efficiency [35]. Due to the high sensitivity of gas chromatography (GC) and high resolution of mass spectrometry (MS), the GC–MS technique is capable of identifying compounds [36]. Additionally, GC–MS allows for the simultaneous separation, identification, and quantification of target analytes, making it widely applicable for the determination of complex mixtures [37].

In this study, we used the pEAQ vector to efficiently express multiple exogenous genes in *N. benthamiana* leaves. We introduced a simple and efficient method for expressing enzymes involved in the biosynthesis pathway of terpenoids in plants and rapidly detected the corresponding products using GC–MS. The efficient co-expression of multiple exogenous genes in tobacco made it possible to construct a heterologous secondary metabolite pathway in *N. benthamiana* and provided an approach for further elucidating the biosynthesis pathway of tanshinones. In this study, by using a cytoplasmic engineering strategy combined with the enhancement of the upstream rate-limiting enzyme HMGR, we found that *N. benthamiana* can produce the key precursor of tanshinones, miltiradiene, at a level of 0.74 mg/g·FW, significantly improving the synthesis efficiency of miltiradiene in tobacco. Furthermore, this study highlights the potential of tobacco plants as alternative platforms for tanshinone production and lays the foundation for further elucidating the functions of downstream catalytic enzymes in the tanshinone biosynthesis pathway.

## 2. Materials and Methods

### 2.1. Plant Material and Growth Conditions

*Nicotiana benthamiana* seeds were sown in pots containing plant seedling culture medium. The cultivation conditions were as follows: temperature of 25 °C, light duration of 16 h, regular watering to ensure the moisture of the culture medium, and selection of 5–6-week-old *N. benthamiana* plants with similar growth status as the injection materials for agrobacterium tumefaciens.

### 2.2. Construction of Plant Transformation Vectors

The cDNA sequences of the *SmGGPPS* (GenBank: FJ643617.1), *SmCPS* (GenBank: EU003997.1), *SmKSL* (GenBank: EF635966.2), and *SmHMGR* (GenBank: EU680958.1) genes were downloaded from the NCBI website. Snapgene 4.3.6 was used to design the primers (Table S1) for these four genes. Using *S. miltiorrhiza* root cDNA as a template, PCR amplification was performed using TransStart^®^ FastPfu DNA Polymerase. The pEAQ vector was cut at 37 °C for 1 h, and the linearized vector and target fragments were recovered using the Zymoclean Gel DNA Recovery Kit. The target fragments were then ligated to the plant expression vector pEAQ using the TianGen EasyGeno Rapid Recombination Kit (single fragment method). The recombinant product was added to *Escherichia coli* DH10B competent cells, gently mixed with a pipette, and subjected to a 30 min ice bath, 90 s 42 °C metal bath, and 2 min ice bath. Then, 700 μL of Luria-Bertani (LB) liquid medium was added, and the mixture was incubated at 37 °C with shaking at 200 rpm for 45 min. After centrifugation at 5000 rpm for 3 min, the supernatant was removed, leaving approximately 100 μL of medium. The bacterial cells were resuspended by gentle pipetting and spread onto LB solid medium containing 50 mg/L kanamycin (Kan). The plates were inverted and incubated at 37 °C overnight. After the growth of *E. coli* carrying the recombinant plasmids, positive colonies were selected and sequenced. The positive colonies were then picked and cultured in 2 mL of LB liquid medium containing 50 mg/L Kan at 37 °C with shaking at 200 rpm for 1 d. Plasmid extraction was performed using the TianGen Plasmid Mini Extraction Kit, resulting in the recombinant plasmids pEAQ-SmGGPPS, pEAQ-SmCPS, pEAQ-SmKSL, and pEAQ-SmHMGR. The nucleotide sequences of SmGGPPS, SmCPS, and SmKSL were translated into amino acid sequences using the website (https://web.expasy.org/translate) accessed on 1 January 2020. The SignalP 4.0 website was used to predict the protein signal peptides, and the corresponding nucleotide sequences were removed during primer design. The same method was used to obtain the recombinant plasmids pEAQ-SmtGGPPS, pEAQ-SmtCPS, and pEAQ-SmtKSL. Green fluorescent protein (GFP) was added to the C-terminal of the above recombinant plasmids, resulting in pEAQ-SmGGPPS-GFP, pEAQ-SmCPS-GFP, pEAQ-SmKSL-GFP, pEAQ-SmtGGPPS-GFP, pEAQ-SmtCPS-GFP, and pEAQ-SmtKSL-GFP. All the above recombinant plasmids were individually transformed into *Agrobacterium tumefaciens* GV3101 competent cells. The recombinant agrobacterial solution was spread onto plates containing 50 mg/L rifampicin, 30 mg/L gentamycin, and 50 mg/L kanamycin, and incubated at 28 °C for 36–48 h. After the growth of *Agrobacterium*, positive colonies were selected by PCR amplification.

### 2.3. Subcellular Localization

*Agrobacterium tumefaciens* GV3101 cells carrying the recombinant plasmids pEAQ-SmGGPPS-GFP, pEAQ-SmCPS-GFP, pEAQ-SmKSL-GFP, pEAQ-SmtGGPPS-GFP, pEAQ-SmtCPS-GFP, and pEAQ-SmtKSL-GFP were added to YEP liquid medium containing the corresponding antibiotics and cultured on a shaker at 28 °C. The *Agrobacterium tumefaciens* bacterial solution was centrifuged at 4000 rpm for 20 min, the supernatant was removed, and the bacterial cells were resuspended in 10 mL of resuspension solution (10 mmol/L MgCl_2_, 10 mmol/L MES, 100 μmol/L AS). After incubation at room temperature for 1 h, the optical density (OD) was measured, and the OD_600_ was adjusted to 0.8–1.0 using the resuspension solution. The activated bacterial suspension was incubated in the dark for 1–2 h. *N. benthamiana* plants at the age of 5–6 weeks were selected, and the *Agrobacterium tumefaciens* infiltration solution carrying the recombinant plasmids was injected into the tobacco leaves. After 48 h of injection, samples were taken and prepared for microscopy. A Leica TCS SP8 confocal laser scanning microscope was used for observation and imaging. The green fluorescent protein (GFP) was excited at a wavelength of 488 nm, and the emission was detected in the range of 500–550 nm. Chloroplast autofluorescence was excited at a wavelength of 590 nm, and the emission was detected in the range of 640–750 nm.

### 2.4. Miltiradiene Production in N. benthamiana

*Agrobacterium* GV3101 cells carrying the recombinant plasmids pEAQ-SmGGPPS, pEAQ-SmCPS, pEAQ-SmKSL, pEAQ-SmtGGPPS, pEAQ-SmtCPS, pEAQ-SmtKSL, and pEAQ-SmHMGR were infiltrated into the *N. benthamiana* leaves by the same method mentioned above. Five pathways were established: ① pEAQ; ② pEAQ-SmGGPPS+pEAQ-SmCPS+pEAQ-SmKSL; ③ pEAQ-SmtGGPPS+pEAQ-SmtCPS+pEAQ-SmtKSL; ④ pEAQ-SmtGGPPS+pEAQ-SmtCPS+pEAQ-SmtKSL+pEAQ-SmHMGR; and ⑤ pEAQ-SmHMGR. The *Agrobacterium* strains carrying the aforementioned plasmids were mixed in a 1:1 ratio according to the pathways. Samples were harvested after 5 d for compound extraction.

### 2.5. Metabolite Extraction

The samples were ground into powder in liquid nitrogen, and 0.5 g of the accurately weighed powder was transferred into a 2 mL centrifuge tube. Then, 1 mL of hexane (containing 10 ng/μL ethyl decanoate) was added to the sample powder for extraction. Three biological replicates were set up. After extraction, the samples were centrifuged at 12,000 rpm for 10 min, and the supernatant was filtered and collected for GC–MS analysis.

### 2.6. GC–MS Analyses of Products

This study improved the method described by Hu, T et al. [31]. Compound analysis was performed using an Agilent 7890A-5975C gas chromatography–mass spectrometry (GC–MS) instrument equipped with a CYCLODEX-B column (30 m × 0.25 mm × 0.25 μm). High-purity helium gas was used as the carrier gas with a flow rate of 1 mL/min, and split injection was not employed. The temperature program was as follows: initial temperature of 50 °C, held for 2 min, followed by a ramp of 5 °C/min to 250 °C, held for 10 min. Electron ionization (EI) was used as the ionization mode, with the ion source temperature set at 250 °C, quadrupole temperature set at 150 °C, electron energy at 70 eV, and scanning range (*m*/*z*) from 50 to 550. Quantitative analysis of products was performed by integrating the peak areas of the total ion current and the internal standard. All analyses were performed with three biological replicates.

### 2.7. Statistical Analysis

Unless otherwise stated, the data presented in this study are the mean ± standard deviation of three biological replicates. Statistical analysis was performed using GraphPad Prism 8.

## 3. Results

### 3.1. Production of Miltiradiene in N. benthamiana

**Figure 2:**
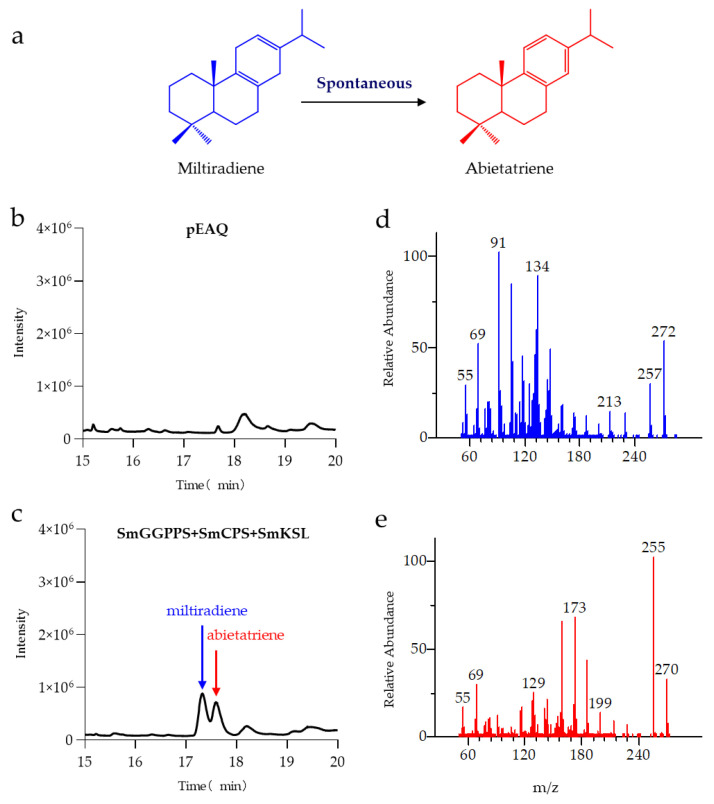
GC–MS detection results of the products from the SmGGPPS+SmCPS+SmKSL pathway. (**a**) Miltiradiene spontaneously generates abietatriene through a self-initiated reaction; (**b**) Chromatogram peaks of the control group with pEAQ empty vector; (**c**) Chromatogram peaks of the experimental group with SmGGPPS+SmCPS+SmKSL; (**d**,**e**) Mass spectrum results corresponding to the chromatogram peaks at 17.6 min (**d**) and 17.8 min (**e**).

The research conducted by Gao, W et al. showcased that SmCPS and SmKSL enzymatically cyclize GGPP to generate miltiradiene [24]. Zi, J et al. found that miltiradiene undergoes aromatization through spontaneous oxidation reactions to form abietatriene [38]. The hydroxylation of miltiradiene at positions C-12 and C-11 to produce ferruginol and 11-hydroxy ferruginol was observed by Guo, J et al. They identified that this biochemical transformation is mediated by CYP76AH1 and CYP76AH3 enzymes derived from *S. miltiorrhiza*. Furthermore, the CYP76AK subfamily, specifically CYP76AK1 from *S. miltiorrhiza*, catalyzes the subsequent oxidation of 11-hydroxy ferruginol, leading to the formation of 11,20-dihydroxy ferruginol and 11,20-dihydroxy sugiol [39,40]. In this study, tobacco leaves were injected with *Agrobacterium* carrying combinations of the *SmGGPPS*, *SmKSL,* and *SmCPS* genes. After 5 d, samples were collected and analyzed by GC–MS. The results, as shown in Figure 2, revealed the appearance of peaks at 17.6 min and 17.8 min in the experimental group compared to the control group. These peaks were preliminarily identified as product peaks, and their mass spectra were compared with the reported spectra of miltiradiene and abietatriene [41]. It was found that the characteristic peaks matched completely. Therefore, the peak at 17.6 min was confirmed as miltiradiene, and the peak at 17.8 min was identified as abietatriene, a spontaneous reaction product of miltiradiene. Our study demonstrates that tobacco can serve as a plant chassis for the production of miltiradiene and abietatriene.

### 3.2. Organelle Localization of Heterologous Proteins

**Figure 3:**
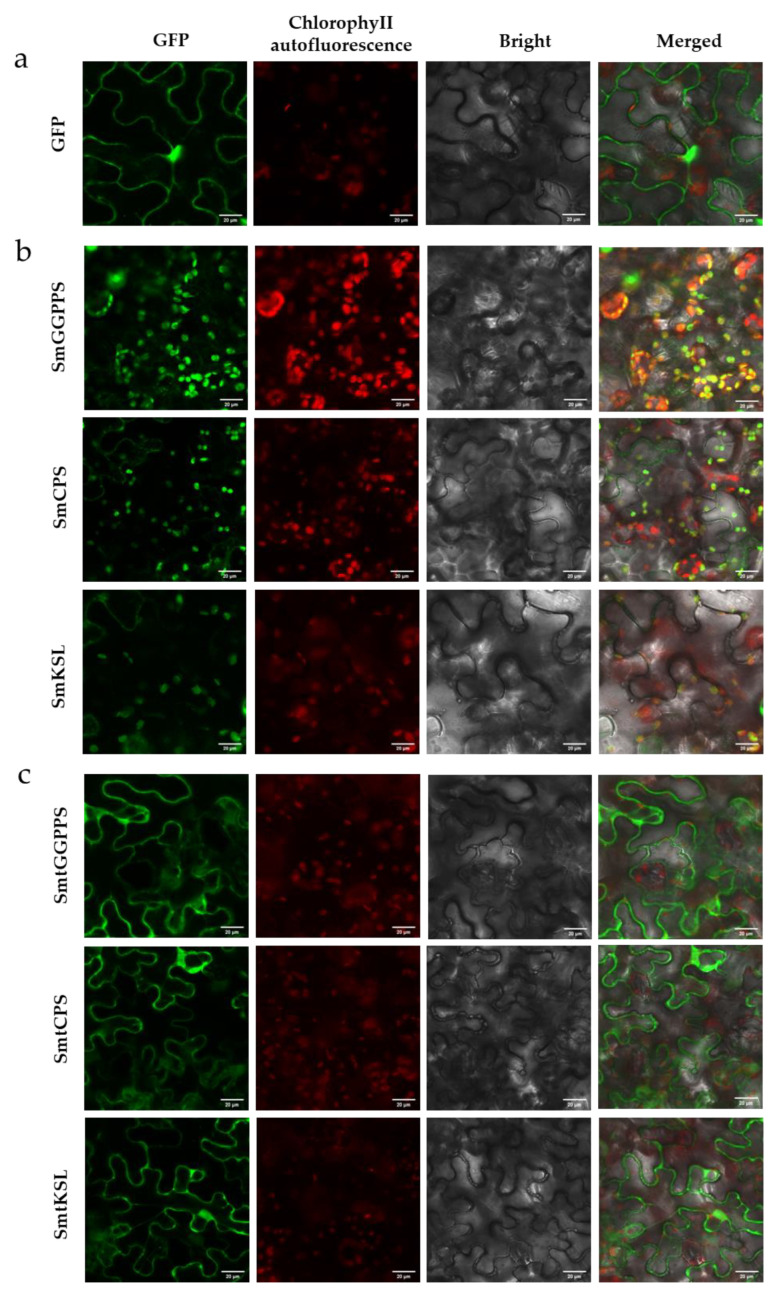
Subcellular localization and retargeting of proteins. (**a**) Fluorescent signal of GFP vector as negative control; (**b**) Subcellular localization results of SmGGPPS-GFP, SmCPS-GFP, and SmKSL-GFP before removal of signal peptides; (**c**) Subcellular localization results of SmtGGPPS-GFP, SmtCPS-GFP, and SmtKSL-GFP after removal of signal peptides. *Agrobacterium* containing the above plasmids were individually injected into the lower epidermis of *N. benthamiana* leaves. Leaf samples were collected 2 d after infiltration and observed using confocal microscopy. Scale bar = 20 μm.

The key enzymes in the upstream biosynthetic pathway of tanshinones are located in plastids, while the downstream CYPs are located in the cytoplasm [42]. To complete the entire biosynthetic pathway and produce the final target compounds, the miltiradiene synthesized in plastids needs to be transported to the cytoplasm, where it undergoes oxidation and modification by a series of CYPs to form various tanshinones. This compartmentalization of different enzymes limits the efficiency of tanshinone synthesis in tobacco [43]. Therefore, in this study, we further modified the synthesis pathway by relocating the enzymes from plastids to the cytoplasm, aiming to reconstruct an efficient cytoplasmic pathway for the biosynthesis of tanshinones. Previous studies have shown that there is a region of RNA encoding a hydrophobic amino acid sequence known as a signal peptide (SP) following the start codon, which is responsible for directing newly synthesized proteins to the secretory pathway [44]. Therefore, we used SignalP 4.0 to predict the signal peptide sequences of SmGGPPS, SmKSL, and SmCPS (Figure S1). The results revealed that the protein sequence of SmGGPPS consisted of 364 amino acids, with the first 53 amino acids being its signal peptide. The protein sequence of SmCPS was composed of 793 amino acids, with the first 76 amino acids serving as its signal peptide. Additionally, the protein sequence of SmKSL comprised 594 amino acids, with 48 amino acids acting as its signal peptide. Subsequently, we performed signal peptide removal for these three proteins. The subcellular localization results are shown in Figure 3. The green fluorescence signal of the pEAQ-GFP vector was distributed on the cell membrane and nucleus (Figure 3a), while the green fluorescence signals of pEAQ-SmGGPPS-GFP, pEAQ-SmCPS-GFP, and pEAQ-SmKSL-GFP before truncation highly overlapped with spontaneous chlorophyll fluorescence (red), indicating that SmGGPPS, SmCPS, and SmKSL were located in chloroplasts (Figure 3b). After removal of the signal peptides, the green fluorescence signals of pEAQ-SmtGGPPS-GFP, pEAQ-SmtCPS-GFP, and pEAQ-SmtKSL-GFP did not overlap with spontaneous chlorophyll fluorescence, indicating successful protein relocalization in the cytoplasm (Figure 3c).

### 3.3. Comparison of Cytoplasmic Pathway and Plastid Pathway Products

**Figure 4:**
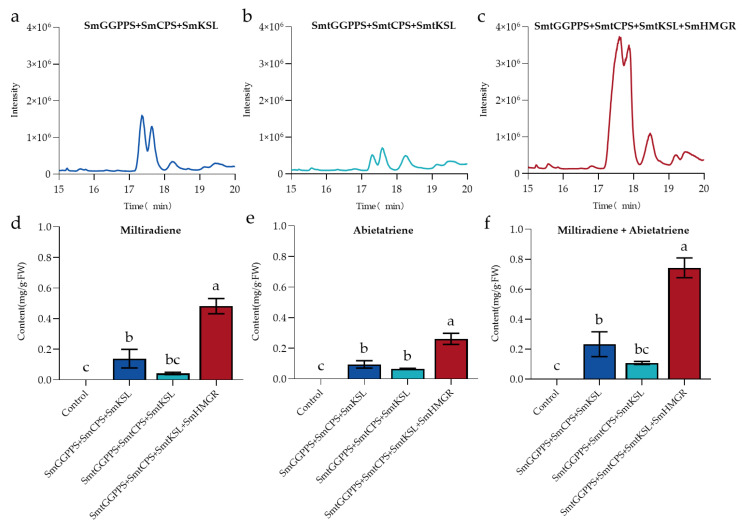
Quantitative analysis of heterologous production of miltiradiene and abietatriene. (**a**–**c**) GC–MS results of SmGGPPS+SmCPS+SmKSL (**a**) SmtGGPPS+SmtCPS+SmtKSL (**b**), and SmtGGPPS+SmtCPS+SmtKSL+SmHMGR (**c**) pathways, respectively; (**d**–**f**) Significance analysis of miltiradiene, abietatriene, and miltiradiene + abietatriene, respectively, by the three constructions above.

The biosynthesis pathways of plant terpenoids can be divided into two routes: the MEP pathway and the MVA pathway [17]. Both pathways can produce terpenoid precursors IPP and DMAPP. The MVA pathway utilizes IPP and DMAPP in the cytoplasm to synthesize C15 sesquiterpenes, C27–29 sterols, C30 triterpenes, and their saponin derivatives, while the MEP pathway utilizes IPP and DMAPP in plastids to synthesize C10 monoterpenes, C20 diterpenes, and C40 tetraterpenes [45]. Tanshinones belong to the C20 diterpenes, with the upstream terpene synthase located in plastids but the downstream CYPs located in the cytoplasm. To overcome the separation caused by the compartmentalization of different enzymes, we reconstructed the biosynthetic pathway of miltiradiene from plastids to the cytoplasm. However, after relocation, the GC–MS results showed that the production of miltiradiene and abietatriene was low, with an average level of only 0.11 mg/g·FW for the total amount, which was approximately half of that obtained through the plastid pathway before modification. This suggested that there was a limited supply of isoprenoid precursors available for diterpene synthases after relocation. We speculate that this may be due to competition between the endogenous terpenoid metabolic pathways in the tobacco cytoplasmic pathway and the exogenous diterpene pathway for terpenoid precursors (IPP and DMAPP), which disrupts the upstream metabolic flow, resulting in lower heterologous production of miltiradiene. Previous studies have shown that HMGR is the rate-limiting enzyme in the MVA pathway [46]. Therefore, we simultaneously enhanced the expression of *SmHMGR* based on the cytoplasmic pathway in order to provide sufficient substrate. The GC–MS results demonstrated a significant improvement in the production of miltiradiene and abietatriene, with a total amount of 0.74 mg/g·FW (Figure 4).

### 3.4. Overexpression of SmHMGR Accelerates Apoptosis in Tobacco Leaves

**Figure 5:**
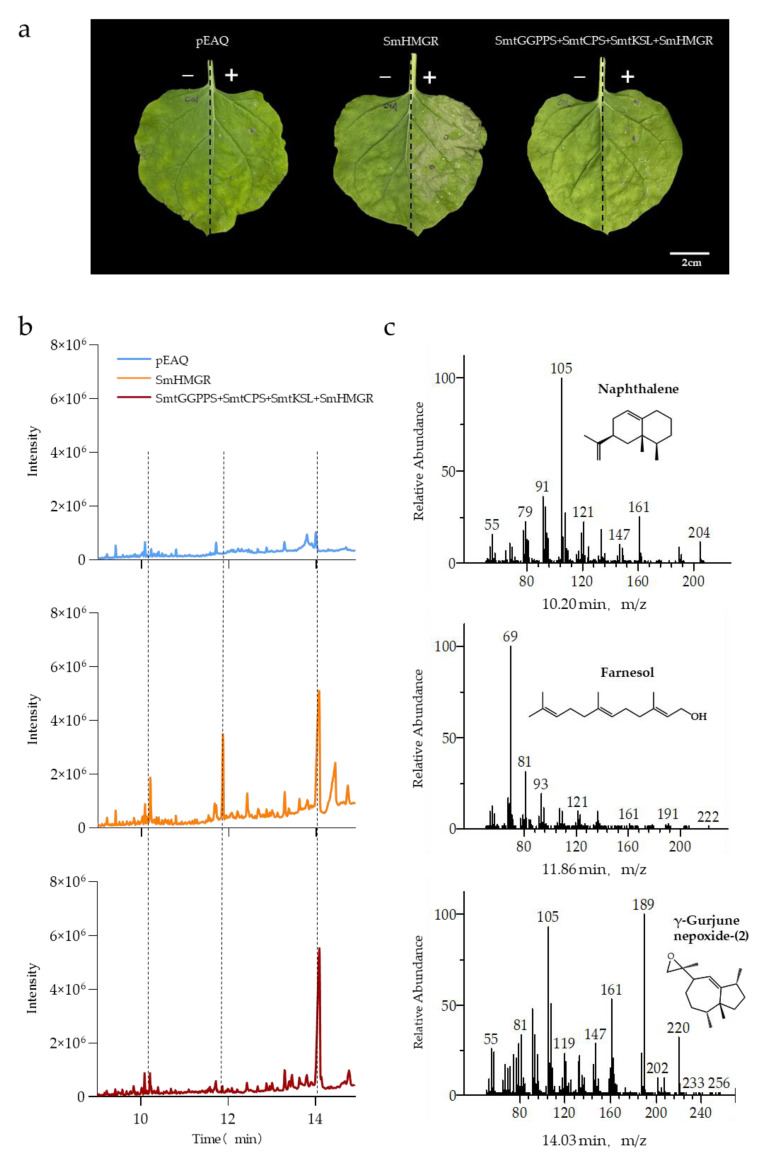
Overexpressing *SmHMGR* causes apoptosis in tobacco leaves. (**a**) The left side of each leaf was kept as a blank control without injection of bacterial solution, while the right side was injected with bacterial solution according to the aforementioned genomic combinations. The photographs were taken on the fifth day after the injection of bacterial solution; (**b**) pEAQ, SmHMGR, and SmtGGPPS+SmtCPS+SmtKSL+SmHMGR GC–MS analysis performed after 5 d post-injection revealed the compounds produced by exogenous proteins; (**c**) GC–MS spectra corresponding to the characteristic peaks in figure (**b**).

In the present study, a comparison of the products obtained from the cytoplasmic pathway reconstruction of miltiradiene (SmtGGPPS+SmtCPS+SmtKSL+SmHMGR) and only SmHMGR overexpression was also performed. After 5 d, severe dehydration and chlorophyll loss was observed in the half with SmHMGR injection, resulting in leaf transparency. By contrast, the symptoms in leaves with the cytoplasmic pathway of miltiradiene containing SmHMGR appeared to be attenuated (Figure 5a). A comparison of the GC–MS results on the 5th day revealed a significant increase in the chromatographic peaks at 10.20 min, 11.86 min, and 14.05 min in tobacco leaves injected with SmHMGR alone, compared to the control injected with pEAQ. The results suggested that these compounds might be related to apoptosis in leaves. To identify these compounds, we searched the mass spectral library, and the compounds were matched to naphthalene (CAS#: 823810-22-6), farnesol (CAS#: 4602-84-0), and γ-gurjunenepoxide-(2) (CAS#: 184705-51-9), respectively (Figure 5c). Further testing of leaves injected with SmtGGPPS+SmtCPS+SmtKSL+SmHMGR revealed a decrease in the chromatographic peaks at 10.20 min and 11.86 min, with a more pronounced decrease in the peak at 11.86 min (Figure 5b). Previous studies have shown that high concentrations of farnesol can be toxic to plant cells [47]. Therefore, we propose that the reason for the better growth of the leaves with the SmtGGPPS+SmtCPS+SmtKSL+SmHMGR combination in contrast to those with SmHMGR alone might be attributed to competition between the exogenous diterpenoid pathway and the sesquiterpennoid farnesol pathway for IPP and DMAPP substrates, resulting in reduced farnesol synthesis and alleviation of cell toxicity in tobacco. 

## 4. Discussion

Terpenoids have been widely applied in the field of medicine and have significant therapeutic effects. Research has shown that diterpenoid compounds such as tanshinone IIA and triptolide have certain therapeutic effects against inflammation, cardiovascular and cerebrovascular diseases, and cancer [48,49]. However, due to limited sources, complex extraction and purification processes, and difficulties in chemical synthesis, large-scale production of terpenoids is challenging [50]. In recent years, with the development of synthetic biology, producing terpenoids and intermediates in heterologous hosts has become an effective alternative method. For example, Christ, B et al. successfully synthesized diosgenin in the tobacco transient transformation system [51], and Hansen, NL et al. elucidated the biosynthetic pathway of triptolide in the *N. benthamiana* system [52]. Li, J et al. achieved successful production of taxol intermediates, namely taxadiene and taxadiene-5α-ol, in *N. benthamiana* [43]. In this study, we also used the tobacco system to successfully produce the key precursor of tanshinones and triptolides, miltiradiene.

The metabolic pathway of terpenoid synthesis is a complex yet promising field with broad applications. By manipulating metabolic flux through metabolic engineering, it is possible to increase crop yield and improve crop quality [53]. *N. benthamiana* is a high biomass non-food crop that is cost-effective, fast-growing, and well-established [54]. Additionally, numerous plant terpene synthases and CYPs have been successfully expressed in this plant, making it an ideal candidate for studying metabolic pathways [55]. Compared to traditional microbial or yeast engineering, plant-based expression systems offer suitable membrane systems, abundant cofactors, and efficient protein folding mechanisms, making them more suitable for heterologous expression of plant enzymes [56]. However, despite these advantages, complex plant cells still have some drawbacks due to their high compartmentalization and the transport mechanisms of metabolites are not yet fully understood, which poses challenges for studying gene functions of relevant enzymes in the secondary metabolism of medicinal plants [57]. In this study, we found that SmGGPPS, SmKSL, and SmCPS possess plastid-targeting peptides, while downstream CYPs are localized in the cytoplasm [58], implying the presence of a physical barrier between the biosynthesis of miltiradiene and subsequent oxidation. Previous studies have shown that, in plant cells, the biosynthesis of terpenoids is divided into plastid (MEP) and cytoplasmic (MVA) pathways, both of which generate the terpenoid precursors IPP and DMAPP [59]. Therefore, we modified the relevant enzymes involved in the production of miltiradiene and successfully produced it in the cytoplasm. However, increasing the biosynthesis of diterpenes in the cytoplasm competes with endogenous sesquiterpenes and triterpenes for substrates, and insufficient supply of IPP or DMAPP affects the yield of miltiradiene. HMGR is recognized as the rate-limiting enzyme in the MVA pathway, and its activity is tightly regulated by the concentrations of IPP and DMAPP, making it a crucial regulatory point in cytoplasmic terpenoid metabolism [60]. Thus, we overexpressed the upstream rate-limiting enzyme *SmHMGR* gene in the cytoplasmic pathway, resulting in an increase in the yield of miltiradiene from 0.23 mg/g·FW to 0.74 mg/g·FW. Miltiradiene is an important intermediate for many abietane-type diterpenoids, and increasing its production provides sufficient precursor material for downstream biosynthesis of active metabolites such as tanshinone, providing a great platform for studying downstream enzyme activity. 

In addition, an interesting phenomenon was also observed in our study, which is the accelerated apoptosis in tobacco leaves when SmHMGR was overexpressed alone. Previous studies have shown that the content of terpenoids is highest in the aging stage of tobacco, followed by that of alkanes, and the expression of the *HMGR* gene is significantly upregulated during aging [61]. Therefore, we hypothesize that the overexpression of SmHMGR might increase the content of apoptosis-related terpenoid compounds, leading to accelerated apoptosis in leaves. GC–MS analysis of tobacco leaf extracts revealed that the overexpression of SmHMGR increased the content of compounds such as naphthalene, farnesol, and γ-gurjunenepoxide-(2). Previous studies have shown that farnesol induces cell toxicity and plant cells can detoxify it by redirecting it to metabolic pathways such as sterol biosynthesis [62]. However, the detoxification capacity is limited, and cells may fail to be protected when it exceeds a threshold. This is consistent with the phenomenon observed in our study, where the introduction of the diterpene synthesis pathway in the cytoplasm diverted the metabolism of terpenoid compounds, thereby partially delaying the apoptosis caused by SmHMGR. The toxicity of naphthalene and γ-gurjunenepoxide-(2) in tobacco cells remains to be explored.

## 5. Conclusions

In summary, this study successfully utilized tobacco to produce miltiradiene and significantly increased its yield from 0.23 mg/g·FW to 0.74 mg/g·FW using a cytoplasmic engineering strategy. This not only demonstrated the feasibility of tobacco as a host plant for the production of natural products but also provided more precursor materials for the study of downstream CYP functions in tanshinone biosynthesis. Furthermore, it was observed that the overexpression of SmHMGR alone accelerated apoptosis in leaves, while co-expression of SmtGGPPS+SmtCPS+SmtKSL with SmHMGR alleviated the apoptosis-related symptoms. It is speculated that this may be due to the diversion of a portion of the metabolic flux in the modified cytoplasmic pathway, leading to a reduction in the synthesis of apoptosis-related compounds and thereby mitigating the apoptosis-related symptoms caused by SmHMGR. This study proposes an alternative platform for microbial systems that can be used for synthetic biology research on tanshinones. Importantly, using plant systems to synthesize heterologous compounds has advantages in terms of safety, environmental friendliness, and cost-effectiveness. Although the production of miltiradiene in the tobacco system is not as high as in the yeast system, this system is of great significance for studying the function of downstream CYPs in the tanshinone biosynthesis pathway. With the successful biosynthesis of miltiradiene in plant cells, we are looking forward to the convenient expression of downstream CYPs in a plant system, which will help establish a rapid screening platform for elucidating the tanshinone biosynthesis pathway. In conclusion, this study demonstrates the heterologous synthesis of the key intermediate, miltiradiene, in tobacco, taking an important step towards studying tanshinone biosynthesis in plant systems and laying a theoretical foundation for the future use of synthetic biology in the heterologous production of other natural medicinal compounds in plants. 

## Data Availability

The data presented in this study are available in the main article and the Supplementary Materials.

## Supplementary Materials

The following supporting information can be downloaded at: https://www.mdpi.com/article/10.3390/metabo13121188/s1, Table S1: Primer sequences for plasmid construction; Figure S1: The signal peptides of SmGGPPS, SmCPS, and SmKSL.
