# Supplementary material for: Miltiradiene Production by Cytoplasmic Metabolic Engineering in Nicotiana benthamiana
Source: Metabolites. 2023 Dec 6;13(12):1188. doi: 10.3390/metabo13121188 (PMC10745046; doi:10.3390/metabo13121188)
Supplement: Supplementary file 1 [file metabolites-13-01188-s001.zip › metabolites-2730740-supplementary.pdf]

Table S1 Primers sequence for plasmid construction.

Primers	Sequences (5' to 3')
SmGGPPS-F	TTCTGCCCAAATTCGCGACCGGTATGAGATCTATGAATCTGGTGGATG
SmGGPPS-R	GAGTTAAAGGCCTCGAGTCAGTTCTGCCTATGTGCAATGTAATCG
SmtGGPPS-F	TTCTGCCCAAATTCGCGACCGGTATGGCCGTGCTCACCGGCGAGGAGG
Sm(t)GGPPS-GFP-R	TCTACCATGCTTCCGCTTCCGTTCTGCCTATGTGCAATGTAATCG
SmCPS-F	TTCTGCCCAAATTCGCGACCGGTATGGCCTCCTTATCCTCTACAATCC
SmCPS-R	GAGTTAAAGGCCTCGAGTCACGCGACTGGCTCGAAAAGCACT
SmtCPS-F	TTCTGCCCAAATTCGCGACCGGTATGCAAGGCCATGATGCGGTGAAGA
Sm(t)CPS-GFP-R	TCTACCATGCTTCCGCTTCCCGCGACTGGCTCGAAAAGCACT
SmKSL-F	TTCTGCCCAAATTCGCGACCGGTATGTCGCTCGCCTTCAACCCGGCAG
SmKSL-R	GAGTTAAAGGCCTCGAGTCATTTCCCTCTCACATTATTAGCT
SmtKSL-F	TTCTGCCCAAATTCGCGACCGGTATGAACCTTACTACAACAGATTTGA
Sm(t)KSL-GFP-R	TCTACCATGCTTCCGCTTCCTTTCCCTCTCACATTATTAGCT
GFP-F	GGAAGCGGAAGCATGGTAGATCTGACTAGTAAAGGAG
GFP-R	GAGTTAAAGGCCTCGAGTCAGCTAGCTTTGTATAGTTCATCCATG
SmHMGR-F	TTCTGCCCAAATTCGCGACCGGTATGGATATCCGCCGGAGGCCAGCCC
SmHMGR-R	GAGTTAAAGGCCTCGAGTCAGGAGCCAATCTTCGTGATGTCCCTG

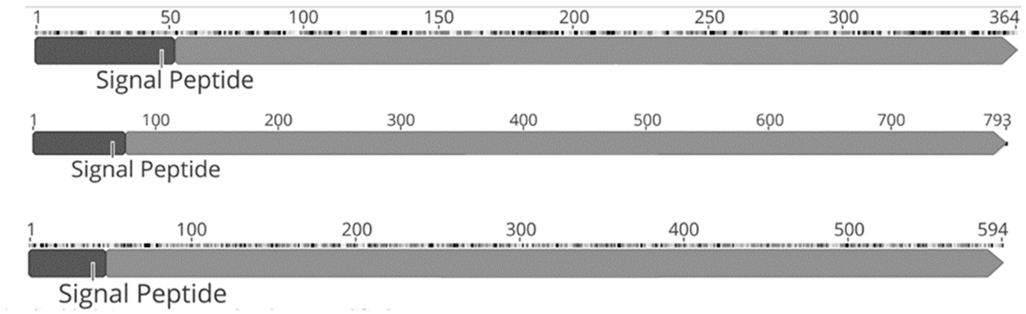

Figure S1 The signal peptides of SmGGPPS, SmCPS and SmKSL.
